# Hepatitis B and C virus seroprevalence, Burkina Faso: a cross-sectional study

**DOI:** 10.2471/BLT.18.208603

**Published:** 2018-08-29

**Authors:** Nicolas Meda, Edouard Tuaillon, Dramane Kania, Adama Tiendrebeogo, Amandine Pisoni, Sylvie Zida, Karine Bollore, Isaïe Medah, Didier Laureillard, Jean Pierre Moles, Nicolas Nagot, Koumpingnin Yacouba Nebie, Philippe Van de Perre, Pierre Dujols

**Affiliations:** a Ministère de la Santé, Ouagadougou, Burkina Faso.; b Pathogenesis and Control of Chronic Infections, Université de Montpellier, U 1058, 60 rue de Navacelles Montpellier, 34394 cedex 5, France.; c Centre Muraz, Bobo-Dioulasso, Burkina Faso.; d Institut National de la Statistique et de la Demographie, Ministère de l’Economie, des Finances et du Développement, Ouagadougou, Burkina Faso.; e Pathogenesis and Control of Chronic Infections, Centre Hospitalier Universitaire de Montpellier, Montpellier, France.; f Pathogenesis and Control of Chronic Infections, Centre Hospitalier Universitaire de Nîmes, Nîmes, France.; g Pathogenesis and Control of Chronic Infections, Institut National de la Santé et de la Recherche Médicale, Montpellier, France.; h Centre National de Transfusion Sanguine, Ouagadougou, Burkina Faso.

## Abstract

**Objective:**

To estimate population-wide hepatitis B and C seroprevalence using dried blood spot samples acquired for human immunodeficiency virus (HIV) surveillance as part of the 2010–2011 Demographic and Health Survey in Burkina Faso.

**Methods:**

We used the database acquired during the multistage, clustered, population-based survey, in which 15 377 participants completed questionnaires and provided dried blood spot samples for HIV testing. We extracted sociodemographic and geographic data including age, sex, ethnicity, education, wealth, marital status and region for each participant. We performed hepatitis B and C assays on 14 886 HIV-negative samples between March to October 2015, and calculated weighted percentages of hepatitis seroprevalence for each variable.

**Findings:**

We estimated seroprevalence as 9.1% (95% confidence interval, CI: 8.5–9.7) for the hepatitis B surface antigen and 3.6% (95% CI: 3.3–3.8) for hepatitis C virus antibodies, classifying Burkina Faso as highly endemic for hepatitis B and low-intermediate for hepatitis C. The seroprevalence of hepatitis was higher in men than in women, and varied significantly for both with age, education, ethnicity and region. Extremely high HCV-Ab seroprevalence (13.2%; 95% CI: 10.6–15.7) was identified in the Sud-Ouest region, in particular within the youngest age group (15–20 years), indicating an ongoing epidemic.

**Conclusion:**

Our population-representative hepatitis seroprevalence estimates in Burkina Faso advocate for the inclusion of hepatitis serological tests and risk factor questionnaire items in future surveys, the results of which are crucial for the development of appropriate health policies and infection control programmes.

## Résumé

**Objectif:**

Estimer la séroprévalence du virus de l'hépatite B et de l'hépatite C dans la population au moyen de gouttes de sang séché prélevées pour la surveillance du virus de l'immunodéficience humaine (VIH) dans le cadre de l'enquête démographique et sanitaire réalisée en 2010–2011 au Burkina Faso.

**Méthodes:**

Nous avons utilisé la base de données constituée durant l'enquête en grappes à plusieurs degrés menée auprès de la population, au cours de laquelle 15 377 participants ont rempli des questionnaires et fourni des échantillons de gouttes de sang séché pour le dépistage du VIH. Pour chaque participant, nous en avons extrait des données sociodémographiques et géographiques, concernant notamment l'âge, le sexe, l'origine ethnique, le niveau d'éducation, le niveau de richesse, la situation matrimoniale et la région. Nous avons effectué des tests de l'hépatite B et de l'hépatite C sur 14 886 échantillons séronégatifs pour le VIH entre mars et octobre 2015, et nous avons calculé des pourcentages pondérés de la séroprévalence de l'hépatite pour chaque variable.

**Résultats:**

Nous avons estimé la séroprévalence à 9,1% (intervalle de confiance de 95%, IC: 8,5–9,7) pour l'antigène de surface de l'hépatite B et à 3,6% (IC 95%: 3,3–3,8) pour les anticorps du virus de l'hépatite C, ce qui classait le Burkina Faso comme zone de forte endémie de l'hépatite B et d'endémie faible à intermédiaire de l'hépatite C. La séroprévalence de l'hépatite était plus élevée chez les hommes que chez les femmes et variait fortement, quel que soit le sexe, en fonction de l'âge, du niveau d'éducation, de l'origine ethnique et de la région. Une séroprévalence extrêmement élevée d'anticorps anti-VHC (13,2%; IC 95%: 10,6–15,7) a été relevée dans la région Sud-Ouest, en particulier dans la tranche d'âge la plus basse (15–20 ans), ce qui indiquait une épidémie en cours.

**Conclusion:**

D'après nos estimations de la séroprévalence de l'hépatite représentatives de la population du Burkina Faso, il serait bon d'inclure des tests sérologiques de l'hépatite et des questions sur les facteurs de risque dans les prochaines enquêtes, dont les résultats seront cruciaux pour l'élaboration de politiques de santé appropriées et de programmes de lutte contre les infections.****

## Resumen

**Objetivo:**

estimar la seroprevalencia de la hepatitis B y C en toda la población con muestras de sangre seca obtenidas para el control del virus de la inmunodeficiencia humana (VIH) como parte de la Encuesta demográfica y de salud entre 2010 y 2011 en Burkina Faso.

**Métodos:**

Se utilizó la base de datos adquirida durante la encuesta multietápica, agrupada y basada en la población, en la que 15 377 participantes completaron cuestionarios y proporcionaron muestras de sangre seca para la prueba del VIH. Se extrajeron datos sociodemográficos y geográficos que incluyen edad, sexo, origen étnico, educación, nivel económico, estado civil y región de cada participante. Se realizaron pruebas de hepatitis B y C en 14 886 muestras VIH negativas entre marzo y octubre de 2015, y se calcularon porcentajes ponderados de seroprevalencia de hepatitis para cada variable.

**Resultados:**

Se estimó la seroprevalencia como el 9,1 % (intervalo de confianza 95 %, IC: 8,5-9,7) para el antígeno de superficie de la hepatitis B y el 3,6 % (IC 95 %: 3,3-3,8) para anticuerpos del virus de la hepatitis C, clasificando a Burkina Faso como una zona altamente endémica para la hepatitis B y bajo intermedio para la hepatitis C. La seroprevalencia de la hepatitis fue mayor en los hombres que en las mujeres y varió significativamente para ambos con la edad, la educación, la etnia y la región. Se identificó una seroprevalencia de HCV-Ab extremadamente alta (13,2 %; IC del 95 %: 10,6-15,7) en la región suroeste, en particular dentro del grupo de edad más joven (15-20 años), lo que indica una epidemia en curso.

**Conclusión:**

Nuestras estimaciones de seroprevalencia de hepatitis en poblacionales de Burkina Faso abogan por la inclusión de pruebas serológicas de hepatitis y elementos sobre factores de riesgo en encuestas futuras, cuyos resultados son cruciales para el desarrollo de políticas de salud apropiadas y programas de control de infecciones.

## ملخص

**الغرض:**

تقدير الانتشار المصلي لفيروسي الالتهاب الكبدي "ب" و"جـ" على نطاق واسع باستخدام عينات بقع دم مجففة تم الحصول عليها من أجل مراقبة فيروس العوز المناعي البشري (HIV) كجزء من المسح الديموغرافي والصحي للفترة من 2010 إلى 2011 في بوركينا فاصو.

**الطريقة:**

استخدمنا قاعدة البيانات التي تم الحصول عليها خلال المسح العنقودي متعدد المراحل، القائم على السكان، حيث أكمل 15377 مشاركًا الاستبيانات وقدموا عينات من بقع دم مجففة لاختبار فيروس العوز المناعي البشري. استخرجنا البيانات الاجتماعية والديموغرافية الجغرافية بما في ذلك العمر والجنس والعرق والتعليم والثروة والحالة الاجتماعية والمنطقة لكل مشارك. أجرينا فحوصات الالتهاب الكبدي "ب" و "جـ" على 14886 عينة سلبية لفيروس العوز المناعي البشري ما بين مارس/آذار وأكتوبر/تشرين أول 2015، واحتسبنا النسب المئوية لمعدل الانتشار المصلي للالتهاب الكبدي لكل متغير.

**النتائج:**

لقد قدرنا الانتشار المصلي بـ 9.1٪ (فاصل الثقة 95٪، 8.5 إلى 9.7) لمستضد الالتهاب الكبدي "ب" السطحي، و3.6٪ (فاصل الثقة 95٪: 3.3 إلى 3.8) للأجسام المضادة لفيروس الالتهاب الكبدي "جـ"، مع تصنيف بوركينا فاصو على أنها مستوطنة منطقة موبوءة للغاية بالالتهاب الكبدي الوبائي "ب"، وموبوءة بشكل متوسط إلى ضعيف بالالتهاب الكبدي "جـ". كان الانتشار المصلي للالتهاب الكبدي أعلى لدى الرجال منه لدى النساء، وتفاوت بشكل كبير بينهما بالنسبة لكل من العمر والتعليم والعرق والمنطقة. تم تحديد نسبة عالية للغاية من الانتشار المصلي للالتهاب الكبدي "أ" و"ب" و"جـ" (13.2٪؛ بفاصل ثقة 95٪: 10.6 إلى 15.7) في المنطقة الموجودة جنوب غرب البلاد، ولا سيما ضمن الفئة العمرية الأصغر (15 إلى 20 سنة)، مما يشير إلى استمرار الوباء.

**الاستنتاج:**

إن ****تقديراتنا للانتشار المصلي للالتهاب الكبدي على مستوى البلاد في بوركينا فاصو، يؤيد تضمين اختبارات علم الأمصال للالتهاب الكبدي، وعناصر استبيان عامل الخطر في المسوح المستقبلية، والتي تعتبر نتائجها حاسمة لتطوير سياسات صحية مناسبة وبرامج لمكافحة العدوى. ****

## 摘要

**目的:**

旨在采用 2010–2011 年布基纳法索人口和健康调查中人类免疫缺陷病毒 (HIV) 监测所获取的干血斑样本来估计全人口范围内乙型肝炎和丙型肝炎血清阳性率。

**方法:**

我们采用了这项基于人口的多级整群调查所获得的数据资料，在这项调查中，共有 15377 名参与者完成了调查问卷并提供了用于 HIV 检测的干血斑样本。我们搜集了每位参与者的社会人口数据和地理信息数据，包括年龄、性别、种族、教育、财富、婚姻状况和地区。我们在 2015 年 3 月至 10 月期间对 14886 例 HIV 阴性样本开展了乙型肝炎和丙型肝炎检测，并计算了各个变量的肝炎血清阳性率加权百分比。

**结果:**

我们估算的乙型肝炎表面抗原血清阳性率为 9.1%（95% 置信区间，CI：8.5–9.7），丙型肝炎病毒抗体的血清阳性率为 3.6%（95% 置信区间，CI：3.3–3.8），布基纳法索被划分为乙型肝炎高患病率和丙型肝炎低中等患病率国家。男性肝炎血清阳性率高于女性，并且在不同年龄、教育、种族和地区之间存在显著差异。西南地区的丙型肝炎抗体血清阳性率（为 13.2%；95% 置信区间，CI：10.6–15.7）极高，尤其是在最年轻的年龄段（15-20 岁），这表明目前疾病仍在蔓延。

**结论:**

我们在布基纳法索的典型群体肝炎血清阳性率估计值表明我们应该将肝炎阳性率检测和风险因素问卷调查纳入今后的调查中，其结果对制定合理的医疗卫生政策和感染控制方案至关重要。

## Резюме

**Цель:**

Оценка серораспространенности вирусов гепатита B и C среди населения с использованием образцов сухой капли крови, полученных в рамках надзора за распространением вируса иммунодефицита человека (ВИЧ), проводившегося в ходе демографического обследования и обследования состояния здоровья населения Буркина-Фасо в 2010–2011 гг.

**Методы:**

Использовалась база данных, созданная во время проведения многоступенчатого кластерного обследования населения, в котором 15 377 участников заполнили опросники и предоставили образцы сухой капли крови для обследования на ВИЧ. Отбирались социально-демографические и географические данные участников, включая возраст, пол, этническую принадлежность, образование, уровень благосостояния, семейное положение и регион проживания каждого из участников. В период с марта по октябрь 2015 г. было проведено тестирование на наличие гепатита B и C 14 886 ВИЧ-отрицательных образцов, после чего были рассчитаны взвешенные процентные доли серораспространенности гепатита для каждой переменной.

**Результаты:**

По нашей оценке, серораспространенность составляет 9,1% (95%-й доверительный интервал, ДИ: 8,5–9,7) для поверхностного антигена гепатита B и 3,6% (95%-й ДИ: 3,3–3,8) для антител к вирусу гепатита C. Соответственно, Буркина-Фасо попадает в категорию стран с высокой распространенностью вируса гепатита B и низким и средним уровнем распространенности вируса гепатита C. Серораспространенность гепатита выше среди мужчин, чем среди женщин, и значительно различается в обеих группах в зависимости от возраста, образования, этнической принадлежности и региона проживания. Чрезвычайно высокий показатель серораспространенности антител к вирусу гепатита C (13,2%; 95%-й ДИ: 10,6–15,7) обнаружен в Юго-Западном регионе, в частности среди самой молодой группы населения (15–20 лет), что указывает на продолжающуюся эпидемию.

**Вывод:**

Проведенная оценка серораспространенности гепатита среди населения Буркина-Фасо свидетельствует о необходимости включения серологических анализов крови на гепатит и пунктов опросников по оценке факторов риска в будущие исследования, результаты которых имеют огромное значение для разработки соответствующих политик в области здравоохранения и программ контроля за распространением вирусной инфекции.

## Introduction

Viral hepatitis is a global health challenge worldwide, particularly in low- and middle-income countries.^1^ Hepatitis B virus (HBV) is estimated to affect around 75 million people in Africa, including 1.9 million in Burkina Faso.^2^ HBV is the most frequent cause of acute hepatitis and chronic liver disease.^3^^,^^4^ Despite the introduction of HBV vaccines in the Expanded Programme on Immunization (EPI) in 2006, incidence is increasing.^2^ Further, 10–33 million individuals in West Africa are estimated to be affected by hepatitis C virus (HCV).^5^ These HBV and HCV infections are the main contributors to the hepatocellular carcinoma burden in Africa.^3^^,^^6^

Control of the HCV epidemic is envisioned when the new direct antiviral agents, reported to cure most HCV-infected patients within 12 weeks of treatment,^7^ become available in low-income countries. Precise epidemic knowledge based on reliable nationwide representative surveys^8^ is therefore essential to estimate the number of HCV-infected subjects requiring treatment and to evaluate the efficiency of any future treatment programme. Data from relevant surveys are also required to estimate the number of people living with chronic HBV and therefore the resources required for treatment.^9^

Robust epidemiological studies on viral hepatitis at the national level are however lacking.^8^ Most existing studies were conducted at different periods on specific populations (e.g. pregnant women or blood donors), and do not report estimations of age, sex, or demographic-specific prevalence.^10^^–^^13^ When included in systematic reviews and meta-analyses of population-wide prevalence,^2^^,^^8^^,^^14^^–^^19^ such studies lead to biases and large estimation intervals, and overlook the demographic and geographic heterogeneity of the epidemics.^8^

The Demographic and Health Surveys (DHS) Program^20^ conducts large, multistage, clustered, population-based surveys in low- and middle-income countries. By collecting and analysing accurate and representative data on population and health, including blood sample collection (dried blood spot) and storage on filter papers for human immunodeficiency virus (HIV) testing, one can evaluate the impact of existing health programmes and develop strategies for improvement. Here, we demonstrate how these surveys provide an opportunity for affordable hepatitis testing and epidemiological studies. Using the DHS database and dried blood spot samples acquired from May 2010 to January 2011, we estimate the viral hepatitis B and C seroprevalence in the HIV-negative adult population of Burkina Faso.

## Methods

### Survey

The *Institut National de la Statistique et de la Démographie* conducted the fourth Burkina Faso DHS in 2010.^21^ Following the structure of previous surveys,^20^ the survey aimed to acquire representative data on households, men and women, and adopted a two-stage, stratified clustered design^22^ in determining which households were to be sampled. In the first stage of the survey, 574 administrative demographic zones were selected from the total of 13 989 within Burkina Faso with a probability dependent upon the number of households within that zone. In the second stage, households within selected demographic zones were selected at random with a uniform probability; a total of 14 947 households were included in the survey. Questionnaires were completed by both male and female interviewers recruited by the *Institut National de la Statistique et de la Démographie*.^21^ Interviewers attended a 6-week training course at the institute, where they were educated on the different features of the survey including general methodology, logistics, the subjects covered in the questionnaire and blood sampling procedures.

### Survey subpopulation

Due to their perceived status as main carer, women were considered best placed to provide information on the health of household members and living conditions. All women aged 15–49 years either living in each of the 14 947 households or having stayed there overnight were therefore invited to complete a questionnaire. Among the households, one household out of two within each demographic zone was selected at random (7475 households) for men aged 15–59 years to be interviewed. Agreement to provide a blood sample was sought from all interviewed men and from the interviewed women who were living in the same households as male participants. After signing an informed consent form, a total of 8293 women and 7084 men formed the subpopulation that completed the questionnaire and provided a blood sample.

### Ethics

All procedures performed as part of the study were conducted in accordance with the standards of the Ethics Committee for Health Research of Burkina Faso, while also meeting the principles expressed in the declaration of Helsinki. Free informed consent was obtained from every individual participant in the use of data and dried blood spot samples collected for further research. According to the Burkina Faso survey protocol, all data records were fully anonymous without any possibility of participant identification.

### Blood sample processing

As part of the Burkina Faso DHS,^21^ dried blood spot samples (two to five per filter paper; Whatman 903 Protein Saver, Dassel, Germany) obtained by finger prick, were sent to the national blood transfusion centre in individual bags with dessicant and stored at −20 °C. Samples were punched into microtitration plates and analysed for HIV infection using an enzyme-linked immunosorbent assay (Vironostika HIV Uni-Form II plus O, Biomérieux, France). The positive samples and 10% of the negative samples were checked using a recombinant enzyme-linked immunosorbent assay (Enzygnost Anti-HIV 1/2 plus, Dade Behring Marburg GmbH, Germany). All discordant results were tested (InnoLia, Innogenetics, Belgium) once more to give a final result of 160 HIV-positive samples. HIV analysis exhausted a total of 491 samples, leaving 14 886 HIV-negative samples for hepatitis testing.

### Hepatitis serology assays

We performed the hepatitis B and C assays on HIV-negative dried blood spot samples between March and October 2015. We tested for the presence of hepatitis B surface antigen (HBsAg) by eluting a punched disc of 6 mm in diameter overnight at ambient room temperature in 1.5 mL phosphate buffered saline, and using the Monolisa HBsAg Ultra assay (Bio-Rad, Hercules, United States of America). The sensitivity and specificity estimations of the test were 96.0% (95% confidence interval, CI: 77.7–99.8) and 100.0% (95% CI: 97.6–100.0), respectively.^23^^,^^24^ We detected the HCV antibody (HCV-Ab) by eluting a punch of 6 mm diameter overnight at ambient temperature in 2 mL of a buffer (two thirds phosphate buffered saline and one third manufacturer’s diluent), and using the Monolisa HCV AgAb Ultra assay (Bio-Rad, Marnes-la-Coquette, France). The sensitivity and specificity estimations of the test were 95.0% (95% CI: 83.1–99.4) and 100.0% (95% CI: 98.9–100.0), respectively^23^^,^^25^^,^^26^

### Survey data extraction

We extracted several sociodemographic and geographic variables from the survey database, including: sex, age, declared ethnicity, educational achievement (none, primary, secondary or higher), household wealth index quintile (quintile of the nationwide wealth index distribution constructed from household assets scoring), marital status (including living with a common-law partner), residential area (name of region and whether urban or rural setting) and HIV status (positive or negative) according to the dried blood spot sample analysis in 2010. We also extracted the representativeness weighting data calculated by the survey statisticians,^21^ and used these data to estimate population-wide seroprevalence from the survey subpopulation.

### Statistical methods

We performed statistical analyses with SAS software, version 9.2® (SAS Institute Inc., Cary, USA), using procedures for weighted survey samples. We used an adjusted F-test for qualitative variables. We also created the new variable “tested-couple” to describe two participants reported as living as a couple in the same household, who had both been tested for viral hepatitis markers. We considered our results significant for *P*-values ≤ 0.05, and present results with their 95% CI.

As our study sample contained only HIV-negative subjects, we conducted a complementary analysis to approximate the hepatitis prevalence in the overall population of 15 046 survey participants (14 886 HIV-negative and 160 HIV-positive). We allocated the upper limit of the published B and C hepatitis prevalence estimates (i.e. 15% for HBsAg in Burkina Faso^27^ and 9% for HCV-Ab in West Africa^17^) for the HIV-positive population (1.0% prevalence^21^) to these subjects.

## Results

### Sociodemographic data

Of the 14 886 HIV-uninfected subjects (Table 1), the mean age was 31.9 years (95% CI: 31.6–32.3) in men and 28.6 years (95% CI: 28.4–28.9) in women. A total of 10 017 participants (weighted %: 68.4; 95% CI: 67.5–69.2) had no educational achievement, 10 576 (72.1%; 5% CI: 71.2–73.0) were currently married or living with a partner, and 10 189 (72.5%; 95% CI: 71.7–73.2) lived in a rural setting.

**Table 1:** Estimated hepatitis seroprevalence, by sociodemographic and geographic data, Burkina Faso, 2010–2011

Characteristic	No. sampled		HBV-positive			HCV-positive	
			No.	Weighted %^a^ (95% CI)		No.	Weighted %^a^ (95% CI)
All	14 886		1365	9.1 (8.5–9.7)		565	3.6 (3.3–3.8)
Men	6 830		723	10.5 (9.6–11.4)		286	3.9 (3.4–4.5)
Women	8 056		642	7.8 (7.1–8.6)		279	3.2 (2.8–3.7)
**Age group, years**							
15–19	3 010		303	9.6 (8.3–10.8)		87	2.5 (1.9–3.1)
20–24	2 489		256	10.4 (9.0–11.9)		69	2.6 (1.9–3.3)
25–29	2 265		220	9.9 (8.5–11.3)		88	3.7 (2.8–4.6)
30–34	2 105		199	9.5 (8.1–11.0)		87	3.8 (2.9–4.8)
35–39	1 708		155	9.2 (7.6–10.7)		71	4.1 (3.1–5.1)
40–44	1 387		105	7.4 (5.9–9.0)		68	4.7 (3.4–5.9)
45–49	1 181		78	6.2 (4.6–7.7)		54	4.4 (3.1–5.7)
50–54 (men)	422		26	5.4 (3.1–7.8)		22	5.1 (2.8–7.5)
55–59 (men)	319		23	6.4 (3.6–9.3)		19	5.9 (3.1–8.7)
**Education**							
None	10 017		866	8.6 (7.9–9.2)		422	4.0 (3.5–4.4)
Primary	2 492		249	10.1 (8.7–11.6)		96	3.7 (2.9–4.6)
Second or higher	2 377		250	10.1 (8.5–11.7)		47	1.6 (1.0–2.2)
**Wealth index quintile**							
Poorest	2 568		217	8.5 (7.2–9.8)		154	5.4 (4.4–6.4)
Poorer	2 771		262	9.3 (8.1–10.6)		109	3.5 (2.7–4.2)
Middle	2 774		246	8.6 (7.4–9.8)		113	4.4 (3.4–5.4)
Richer	3 042		292	9.2 (7.9–10.4)		123	3.8 (3.0–4.7)
Richest	3 731		348	9.5 (8.2–10.8)		66	1.5 (1.0–2.0)
**Marital status**							
Never married	3 918		411	10.4 (9.2–11.6)		109	2.3 (1.8–2.9)
Currently married	10 576		911	8.5 (7.9–9.1)		437	4.0 (3.5–4.4)
Previously married	392		43	12.0 (7.7–16.2)		19	4.5 (2.3–6.6)
**Geographical setting**							
Urban	4 697		452	9.6 (8.4–10.8)		101	1.8 (1.2–2.3)
Rural	10 189		913	8.9 (8.2–9.5)		464	4.3 (3.8–4.7)
**Region**							
Boucle du Mouhoun	1 309		109	7.9 (6.3–9.5)		41	3.0 (2.0–4.0)
Cascades	963		95	10.1 (8.2–12.0)		57	6.2 (4.6–7.8)
Centre	1 424		132	9.4 (7.6–11.2)		11	1.0 (0.3–1.7)
Centre-Est	1 009		107	10.3 (8.1–12.5)		35	3.8 (2.5–5.1)
Centre-Nord	1 024		89	8.0 (6.1–10.0)		36	3.9 (2.5–5.3)
Centre-Ouest	1 282		126	9.8 (7.9–11.7)		45	3.9 (2.6–5.1)
Centre-Sud	1 016		76	6.8 (5.1–8.6)		16	1.7 (0.8–2.6)
Est	1 186		127	11.0 (8.9–13.1)		41	4.0 (2.8–5.3)
Hauts-Bassins	1 388		125	9.1 (7.3–10.8)		53	3.7 (2.6–4.8)
Nord	1 090		77	6.6 (4.9–8.3)		41	4.0 (2.6–5.3)
Plateau-Central	1 100		91	8.4 (6.6–10.1)		31	3.0 (1.9–4.1)
Sahel	1 003		110	10.6 (8.5–12.7)		25	2.6 (1.5–3.7)
Sud-Ouest	1 092		101	8.9 (7.0–10.8)		133	13.2 (10.6–15.7)
**Ethnicity**							
Bobo	606		49	7.8 (5.5–10.0)		38	6.2 (4.1–8.4)
Dioula	127		11	8.6 (3.1–14.2)		3	2.0 (0.0–4.3)
Fulfulde/Peul	1 157		111	9.2 (7.3–11.1)		34	3.0 (2.0–4.1)
Gourmantche	946		114	12.3 (9.9–14.6)		34	4.1 (2.7–5.5)
Gourounsi	721		71	8.7 (6.4–11.0)		24	3.4 (1.9–5.0)
Lobi	513		39	7.9 (5.3–10.5)		62	11.6 (8.2–15.0)
Mossi	7 659		624	8.2 (7.5–9.0)		178	2.4 (2.0–2.8)
Senoufo	855		102	12.5 (9.9–15.1)		57	5.5 (4.0–7.0)
Touareg/Bella	239		26	9.8 (6.2–13.3)		6	2.1 (0.6–3.7)
Dagara	495		60	11.2 (7.9–14.5)		70	13.2 (9.6–16.7)
Bissa	516		61	11.8 (8.7–14.8)		18	3.9 (1.9–6.0)
Other	1 052		97	8.8 (6.8–10.8)		41	3.6 (2.4–4.9)

CI: confidence interval; HBV: hepatitis B virus; HCV: hepatitis C virus.

^a^ Percentages and CIs are weighted for population-wide representativeness using data calculated by 2010 survey statisticians.

Note: We estimated the seroprevalence from the 2010 Demographic and Health Survey.^21^

### Seroprevalence

Countrywide, seroprevalence was estimated as 9.1% (95% CI: 8.5–9.7) for HBV, 3.6% (95% CI: 3.3–3.8) for HCV and 0.3% (95% CI: 0.2–0.4) for HBV–HCV coinfection.

### HBV

**Fig. 1:**
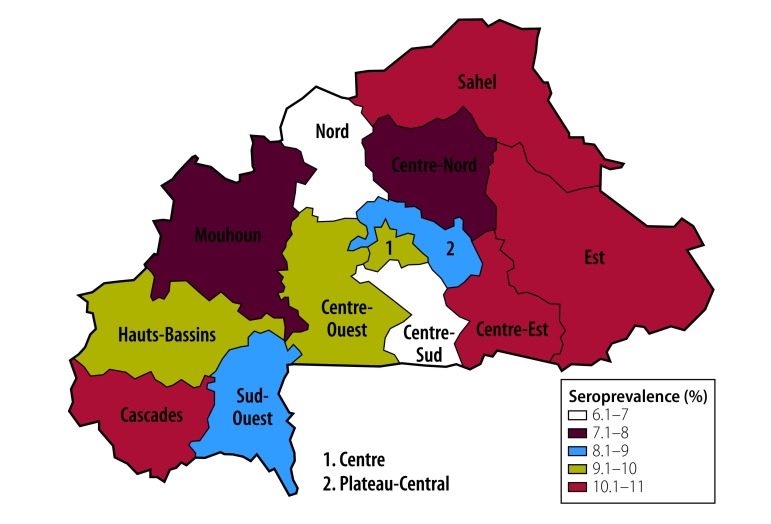
Hepatitis B seroprevalence in Burkina Faso, 2010–2011

Note: We estimated the seroprevalence from the 2010 Demographic and Health Survey.^21^

**Fig. 2:**
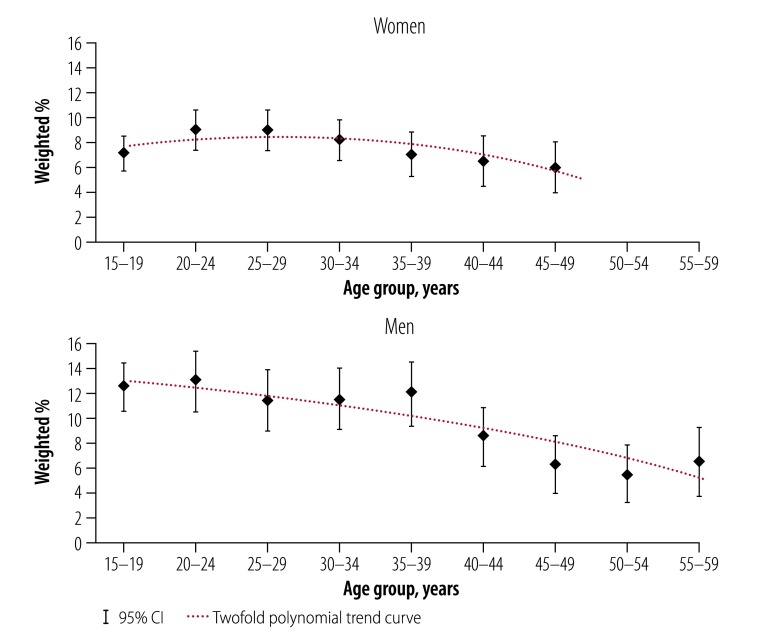
Hepatitis B infection by age and sex, Burkina Faso, 2010–2011

The HBV seroprevalence was significantly higher in men (10.5%; 95% CI: 9.6–11.4) than in women (7.8%; 95% CI: 7.1–8.6). For both men and women, the prevalence varied significantly with age (*P* = 0.0002), level of education (*P* = 0.03), ethnicity (*P* = 0.004) and region of residency (*P* = 0.0029; Fig. 1; Fig. 2 and Table 1). The HBV seroprevalence within the Gourmantche and Senoufo ethnic groups was 12.3% (95% CI: 9.9–14.7) and 12.5% (95% CI: 9.8–15.1), respectively. This is significantly higher than the 8.2% (95% CI: 7.4–9.0) seroprevalence within the predominant Mossi ethnic group, who comprise 51.5% (7659/14 886; 95% CI: 50.4–52.5) of the population in Burkina Faso according to the 2010 survey. In men, lower seroprevalence was observed with increasing age (*P* for trend, 0.003) (Fig. 2). Among couples, the HBV seroprevalence was significantly higher (*P* = 0.02) for those whose partner was infected (11.7%; 95% CI: 8.4–15.1) compared with those whose partner was not infected (8.1%; 95% CI: 7.4–8.9).

CI: confidence interval.

Note: We estimated the seroprevalence from the 2010 Demographic and Health Survey.^21^

### HCV

**Fig. 3:**
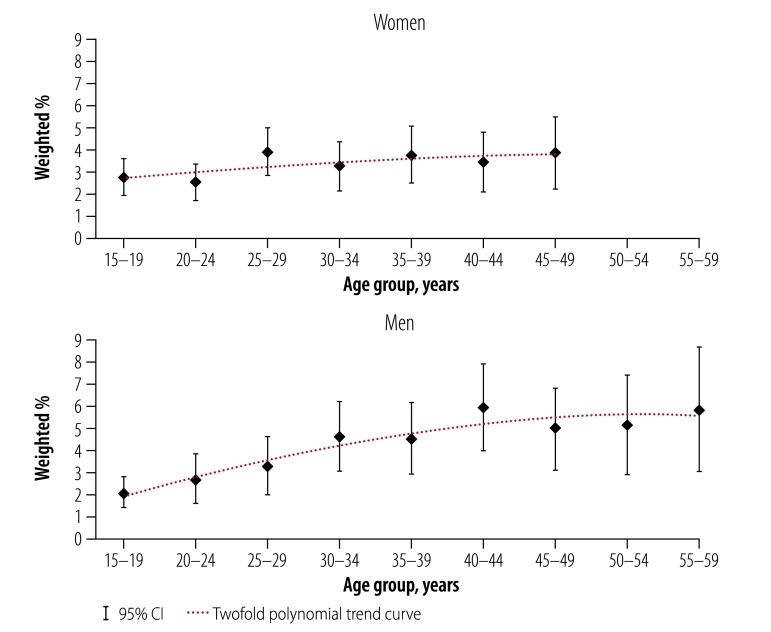
Hepatitis C infection by age and sex, Burkina Faso, 2010–2011

**Fig. 4:**
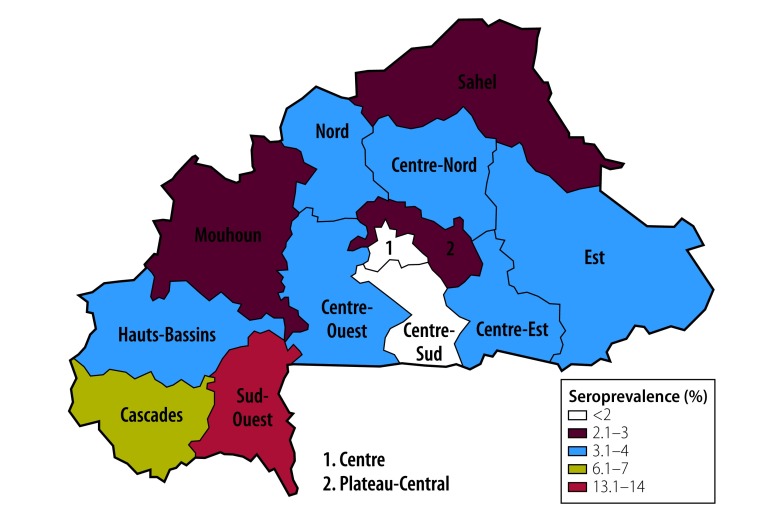
Hepatitis C seroprevalence in Burkina Faso, 2010–2011

Note: We estimated the seroprevalence from the 2010 Demographic and Health Survey.^21^

The seroprevalence of HCV infection was higher in men (3.9%; 95% CI: 3.4–4.5) than in women (3.2%; 95% CI: 2.8–3.7; Table 1). For both men and women, HCV seroprevalence varied with age (*P* = 0.024; Fig. 3), level of education level (*P* < 0.0001), ethnicity (*P* < 0.001) and wealth index quintile (*P* < 0.0001). It varied from 2.4% (95% CI: 2.0–2.8) in the Mossi ethnic group to 11.6% (95% CI: 8.2–15.0) in the Lobi and 13.2% (95% CI: 9.6–16.7) in the Dagara ethnic groups. HCV seroprevalence was significantly (*P* < 0.0001) higher in rural (4.3%; 95% CI: 3.8–4.7) than in urban (1.8%; 95% CI: 1.2–2.3) settings and varied with regions; Centre (1.0%; 95% CI: 0.3–1.7) or Centre-Sud (1.7%; 95% CI: 0.8–2.6) regions had the lowest seroprevalence, while Cascades (6.2%, 95% CI: 4.6–7.8) and Sud-Ouest (13.2%; 95% CI: 10.6–15.7) regions had the highest (Fig. 4). As for HBV, the HCV seroprevalence increased significantly (*P* < 0.0001) for those whose partner was infected (14.6%; 95% CI: 9.4–19.8) compared with those whose partner was not infected (3.7%; 95% CI: 3.2–4.1). In men, the seroprevalence was observed to increase with age (*P* for trend, < 0.001; Fig. 3).

CI: confidence interval.

Note: We estimated the seroprevalence from the 2010 Demographic and Health Survey.^21^

### HCV–HBV coinfection

**Fig. 5:**
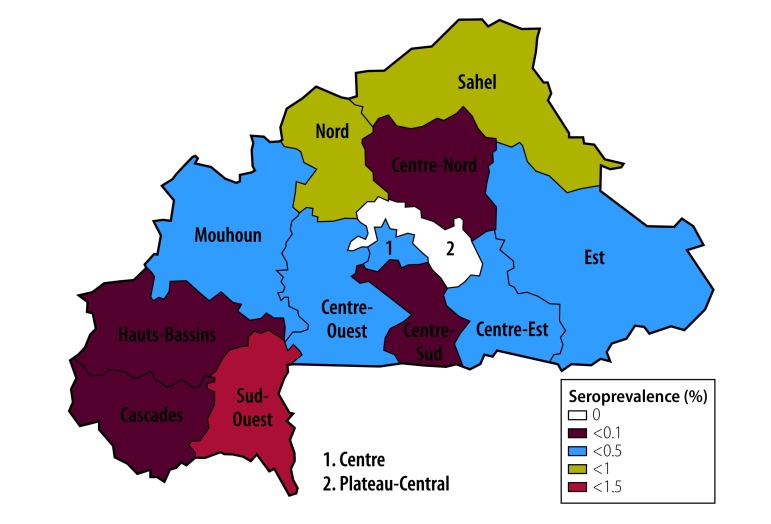
Coinfection of hepatitis B and hepatitis C in Burkina Faso, 2010–2011

Note: We estimated the seroprevalence from the 2010 Demographic and Health Survey.^21^

The seroprevalence of coinfection was lower in women (0.2%; 95% CI: 0.1–0.3) than in men (0.4%; 95% CI: 0.2–0.6), and varied across regions (Fig. 5). Among the population in which HCV-Ab was detected, the seroprevalence of HBsAg was 8.4% (95% CI: 6.4–10.4), significantly (*P* = 0.03) higher in men (10.7%; 95% CI: 7.6–13.7) than in women (6.1%; 95% CI: 3.4–8.7). Among the HBsAg-positive population, the seroprevalence of HCV-Ab was 3.4% (95% CI: 2.4–4.2) countrywide: 4.1% (95% CI: 2.7–5.4) in men and 2.5% (95% CI: 1.4–3.6) in women.

### Complementary analysis

When we included hepatitis seroprevalence estimates for HIV-positive participants, the population-wide seroprevalence of viral hepatitis did not change with a 9.1% HBsAg prevalence (95% CI: 8.5–9.7) and a 3.6% HCV-Ab prevalence (95% CI: 3.3–4.0).

## Discussion

Systematic reviews^2^^,^^8^ have estimated HBV and HCV prevalence in Burkina Faso of 12.0% (95% CI: 11.7–12.4) and 6.1% (95% CI: 1.3–14.2), respectively, ranking the country as one of the most affected sub-Saharan countries.^8^ However, despite meta-analytic designs, these estimations are biased by homogeneity in population sampling. Our calculation of 9.1% HBV seroprevalence (95% CI: 8.5–9.7) and 3.6% HCV seroprevalence (95% CI: 3.3–3.8), based on a representative population sample in terms of sociodemographic and geographic characteristics, are much lower and have narrower CIs. Our data show that Burkina Faso should be classified as highly endemic for HBV (> 8%)^4^ and of low–intermediate prevalence for HCV (3–6%).^17^

Our HBV seroprevalence estimates were roughly uniformly distributed across the geographic regions, but a high degree of regional heterogeneity was observed for HCV; this demonstrates that the assumed epidemiological homogeneity between neighbouring countries used in meta-analyses^8^ is not valid at a national level. In the Sud-Ouest region, populated mostly by the Lobi (45.1%, 492/1092; 95% CI: 41.8–48.3) and Dagara (36.7%, 401/1092; 95% CI: 33.9–39.5) ethnic groups,^21^ HCV seroprevalence is 13.2%; this is close to that of Egypt (14.7%, 1636/11 126),^28^ currently considered the most-affected country in Africa. We also observed a different pattern of HCV age-specific seroprevalence in the Sud-Ouest region: as well as an increasing trend in seroprevalence with age, in common with the countrywide prevalence, a high seroprevalence of 12.9% (95% CI: 7.1–18.7) was estimated in the youngest age group (15–19 years).

Our study has some limitations. The 2010–2011 Burkina Faso DHS focused on the sociodemographic characteristics of the population and the prevalence and risk factors of HIV (e.g. a history of unprotected sex and/or multiple partners, or having undergone unsafe, medically invasive procedures during the previous 12 months). Although these risk factors of hepatitis infection are shared with that of HIV infection,^17^^,^^29^ the survey was not specific enough to measure hepatitis epidemiology; risk factors such as drug use, tattoos or cultural scarification (frequently carried out within the Lobi and Dagara ethnic groups), or hepatitis infection in a relative, were not considered. We therefore limited our analysis of hepatitis seroprevalence to one in terms of sociodemographic parameters only. Future surveys, especially in West Africa, should accommodate the current health challenges of hepatitis epidemiological knowledge and control.

Since the survey was conducted during 2010–2011 and included participants from age 15 years, we cannot investigate the effect of the introduction of the HBV vaccine in the EPI in 2006. However, in countries that have introduced routine infant HBV immunization, prevalence has been observed to fall.^6^

Hepatitis analysis of dried blood spot samples from HIV-negative participants only was a potential selection bias for countrywide prevalence assessment. Nevertheless, with a 1% HIV seroprevalence in Burkina Faso,^21^ our complementary analysis suggests that this bias had no effect on the estimated hepatitis prevalence. The method of determining HIV status within any DHS involves the collection of five dried blood spots per card from each survey participant, sufficient for both HIV (rapid test, confirmation test and viral load) and hepatitis testing if (i) the five blood spots are completely and correctly filled and (ii) sample punches are performed near the margin, allowing three punches per spot.

The HBV and HCV seroprevalence we report here reflect the situation in 2010 and cannot predict the current situation in 2018. However, these data represent a countrywide baseline against which the evolution of the epidemic can be measured in future surveys. From our estimate of HCV prevalence of 3.6%, and considering that 50% of the 15.6 million Burkina Faso population in 2010 was aged 15–59 years^30^ and that infection with hepatitis C resolves spontaneously in about 25% of the infected population,^31^ we estimate that about 210 600 HCV viraemic adults were in need of HCV therapy in 2010.

A final limitation of the study is the ethical conundrum of not returning results to the participants, which is contrary to the 2017 WHO recommendations.^32^ The survey protocol stipulated that the dried blood spot sampling and database would be totally anonymous without any possibility of participant identification; participants could therefore not be informed of HIV-seropositive results. In an attempt to overcome this situation, a list of the nearest health centres and a voucher for a free HIV test were given to each sampled participant. Since our study was conducted in 2015 and nested within the 2010–2011 survey, we were not able to replicate this procedure for hepatitis testing. This limitation does not alter our results or their public health interest, but advocates for free tests and treatments.

Our study benefited from the stratified clustered design of the DHS, a survey design which is similar to that of a national census.^22^ This design results in sample sizes of over 10 000 participants, which is considered the minimum to be wholly representative of the general population for health and demographic data, meeting the requirements for accurate epidemiological results.^5^

An additional benefit of the survey design is the possibility of identifying subregional variations; our discovery of the high level of HCV seroprevalence in the Sud-Ouest region, particularly in the youngest age group (15–20 years), suggests that the epidemic is still ongoing. Further studies are required to identify the transmission routes and understand the specific risk factors associated with this region.^29^^,^^33^^,^^34^

Another strength of our study is its feasibility and low cost. Hepatitis serology testing using dried blood spot samples is now recommended when collecting venous blood specimens is difficult (e.g. in epidemiological studies) and/or when the sample has to be tested away from where it was collected, as is the case in many low- or middle-income countries.^35^^,^^36^ The extra cost per subject for HBV and HCV testing during a survey is approximately 8 United States dollars (US$): US$ 2 for hepatitis serologic reagents (equivalent to a rapid diagnosis test^34^) and US$ 6 for basic laboratory fees.^37^

In conclusion, expanding DHS to include hepatitis testing is both affordable and achievable. Characterizing disease epidemiology and its evolution at nationwide and regional levels in sub-Saharan Africa is crucial for the development of appropriate health policies and infection control programmes.^38^ Our reliable hepatitis B and C seroprevalence data in Burkina Faso, and our identification of an ongoing epidemic in the Sud-Ouest region, advocate for the immediate inclusion of hepatitis serological tests and risk factor questionnaire items in future surveys.
